# Dietary mannose supplementation in phosphomannomutase 2 deficiency (PMM2-CDG)

**DOI:** 10.1186/s13023-020-01528-z

**Published:** 2020-09-22

**Authors:** Roman Taday, Marianne Grüneberg, Ingrid DuChesne, Janine Reunert, Thorsten Marquardt

**Affiliations:** Department of General Pediatrics, University Children’s Hospital Münster, Albert-Schweitzer-Campus 1, 48149 Münster, Germany

**Keywords:** PMM2, Congenital disorder of glycosylation (CDG), Glycoprotein profile, Mannose, Therapy

## Abstract

**Background:**

PMM2-CDG (CDG-Ia) is the most frequent N-glycosylation disorder. While supplying mannose to PMM2-deficient fibroblasts corrects the altered N-glycosylation in vitro, short term therapeutic approaches with mannose supplementation in PMM2-CDG patients have been unsuccessful. Mannose found no further mention in the design of a potential therapy for PMM2-CDG in the past years, as it applies to be ineffective. This retrospective study analyzes the first long term mannose supplementation in 20 PMM2-CDG patients. Mannose was given at a total of 1–2 g mannose/kg b.w./d divided into 5 single doses over a mean time of 57,75 ± 25,85 months. Protein glycosylation, blood mannose concentration and clinical presentation were monitored in everyday clinical practice.

**Results:**

After a mean time period of more than 1 year the majority of patients showed significant improvements in protein glycosylation.

**Conclusion:**

Dietary mannose supplementation shows biological effects in PMM2-CDG patients improving glycosylation in the majority of patients. A double-blind randomized study is needed to examine the role of mannose in the design of a therapy for children with PMM2-CDG in more detail.

## Background

Congenital disorders of glycosylation (CDG) are a steadily growing group of inherited disorders caused by an impaired glycoprotein and -lipid production [1]. The most common type is the N-glycosylation defect caused by phosphomannomutase 2 deficiency (EC 5.4.2.8; PMM2-CDG or CDG-Ia; OMIM 601785) [2]. Patients with PMM2-CDG have a broad and variable spectrum of clinical presentations with psychomotor retardation, muscular hypotonia, failure to thrive, ataxia, epilepsy, strabismus, cerebellar hypoplasia, liver dysfunction, coagulopathy, variable development delay, dysmorphic features, and inverted mammilles; in later stages there may be stroke-like episodes and retinitis pigmentosa, while nerve conduction velocity and tendon reflexes decrease with age [3, 4] Patients suffering from MPI-deficiency (EC 5.3.1.8; CDG-Ib, mannosephosphate isomerase deficiency; OMIM 154550) benefit from a dietary supplementation of mannose, a monosaccharide that bypasses the reduced endogenous Man-6-P (mannose-6-phosphate) production caused by the MPI defect [5] (Fig. S1). In PMM2-CDG utilization of exogenous mannose or endogenous Man-6-P originating from Frc-6-P (fructose-6-phosphate) is impaired, due to the enzyme PMM2 being located downstream their entrance [5–8]. Mannose supplementation in PMM2-CDG should be less or not effective (Fig. S1). However, adding mannose to a culture of PMM2-deficient fibroblasts [9] led to a correction of the glycosylation defects in CDG-fibroblasts [9, 10]. In addition, a hypomorphic PMM2 mouse model showed that mannose could overcome the embryonic lethality of PMM2 offsprings, proving the biological effect of mannose in a PMM2-deficient in vivo model [11]. Short term attempts (up to 6 months) of providing dietary mannose to improve the hypoglycosylation in PMM2-CDG patients followed these findings in vitro, but failed to show improvements [12–15]. Since profound correction of the abnormal isoform pattern took 11 months in MPI-CDG [5], a time frame much longer than the biological half-life of the marker protein transferrin (app. 2 weeks) [16], it could take even more time in PMM2-CDG. To date, mannose applies as utterly ineffective for PMM2-CDG and no causative treatment for this disease is found, yet. This retrospective analysis provides data of positive biochemical effects of a long-term (> 1 year) mannose supplementation in PMM2-CDG.

## Patients and methods

### Patients

Dietary sugar supplementation is standard-of-care in disorders of glycan metabolism in our hospital. Twenty patients (10 female, 10 male) with PMM2-CDG aged ≤1 year - 27 years, who were treated with oral mannose, were analyzed retrospectively (Table S1).

Diagnosis of each patient had previously been confirmed by isoelectric focusing of transferrin [17], by Sanger sequencing of the PMM2 gene and in some patients additionally by measuring PMM2 activity. Data analysis was approved by the local ethics committee (Ethikkommission der Ärztekammer Westfalen-Lippe, No. 2019–199-f-S). All data used in this study were generated from standard clinical follow up visits.

### Mannose supplementation and supervision

Mannose supplementation was done orally or by tube feeding. D-mannose was obtained as a dietary supplement from Nutricia GmbH. Parents were advised to dissolve mannose in water and to give it to their child with or after meals in 5 doses per day. No other nutritional interventions were done. In order to prevent gastrointestinal side effects, mannose was gradually increased over a few weeks to the final dose. If well tolerated, patients had the option to raise the mannose supplementation to a dose higher than 1 g/kg bw/d. Parents and patients were informed orally and in writing about possible side effects. In case of loose stools or flatulence parents were advised to reduce or stop the mannose supplementation until clinical recovery and to raise the dosage again. To control for glycation, HbA1c values were analyzed on a regular basis. Other serum parameters like transaminases, bilirubin, LDH, CK, potassium, sodium, alkaline phosphatase were measured routinely. Depending on the general condition and findings, the consultations were initially held monthly, later every 6 months and annually. Immediate consultation was possible at all times in the event of problems or questions by the parents or patients.

### Mannose assay

D-Mannose serum concentrations were determined by gas chromatography/ mass spectrometry employing trimethylsilyl derivates as described before [18], using perseitol and trehalose as internal standards. Blood samples were taken during routine clinical follow up visits in our out-patient clinic so that there were different periods of time between mannose intake and drawing the blood sample.

### Glycoprotein analyses

Serum or capillary blood samples in heparinized Microvettes® were used for quantification of transferrin isoforms by high performance liquid chromatography (HPLC) using the “CDT in serum- HPLC” kit from Chromsystems (Gräfelfing, Germany) [19]. Reference values of Tf- HPLC have been published earlier [19, 20]: asialo-Tf: below level of detection; monosialo- Tf: below level of detection; disialo- Tf: 1,1 ± 0,72%; trisialo- Tf: 3,76 ± 2,6%; tetrasialo- Tf: 89,84 ± 4,16%; pentasialo- Tf: 6,4 ± 3,8%. The percentage of tetrasialotransferrin indicates the relative amount of tetrasialotransferrin compared to the total transferrin amount, a value commonly used for diagnostics. Untreated patients can have very different amounts of tetrasialotransferrin, a value that has little fluctuation in an individual patient but can be very different from one patient to the other. In order to make patients comparable, the pretreatment tetrasialotransferrin was normalized to 100% so that the relative changes in each patient can be compared.

Isoelectric focusing (IEF) was performed as described before [20–22]. Plasma levels of ATIII, factor IX, protein C and S were determined by routine clinical chemistry. Reference values: AT III: 82–126%; Prot. C act.: 70–140%; Factor XI: > 70%; Protein S: 60–140%.

### Clinical data collection

Medical data of the 20 patients described in this study were retrospectively obtained between 1998 and 2019 from medical reports as well as from caretakers’ reports. Data for motor skills, speech, reading, writing and counting for each patient were raised using different methods including Griffith- Test, MFED (Münchener funktionelle Entwicklungsdiagnostik), developmental steps after Clahsen, physical examination in our ambulance and pediatric neurology as well as from other medical professionals and the parents’ reports. Every patient received physiotherapy (Voijta, Bobath or Galileo) as well as logopedic treatment and ergotherapy.

## Results

### Mannose supplementation and supervision

Mean period of mannose supplementation was 57,75 ± 25,85 months (Table S1). At the time of data collection, 9 patients were still treated with mannose without a long-term interruption. Eleven patients stopped or interrupted the mannose supplementation for different periods of time. Episodes of mild diarrhea and/or flatulence occurred as side effects of mannose ingestion. No other side effects were observed. No patient developed increased HbA1c values as it was described for oral mannose therapy in a MPI-CDG patient [7] (treated patients mean: 4,43 ± 0,7%; reference range: 4,2- 6,1%). A positive response to mannose therapy was defined as increase of tetrasialo-transferrin in Tf-HPLC by 50% of pretreatment levels within 2–3 years of mannose therapy. Probands were divided in “Responders” (*n* = 12) and “Non-responders” (*n* = 8) (Table S1).

### Glycoprotein analyses

**Fig. 1:**
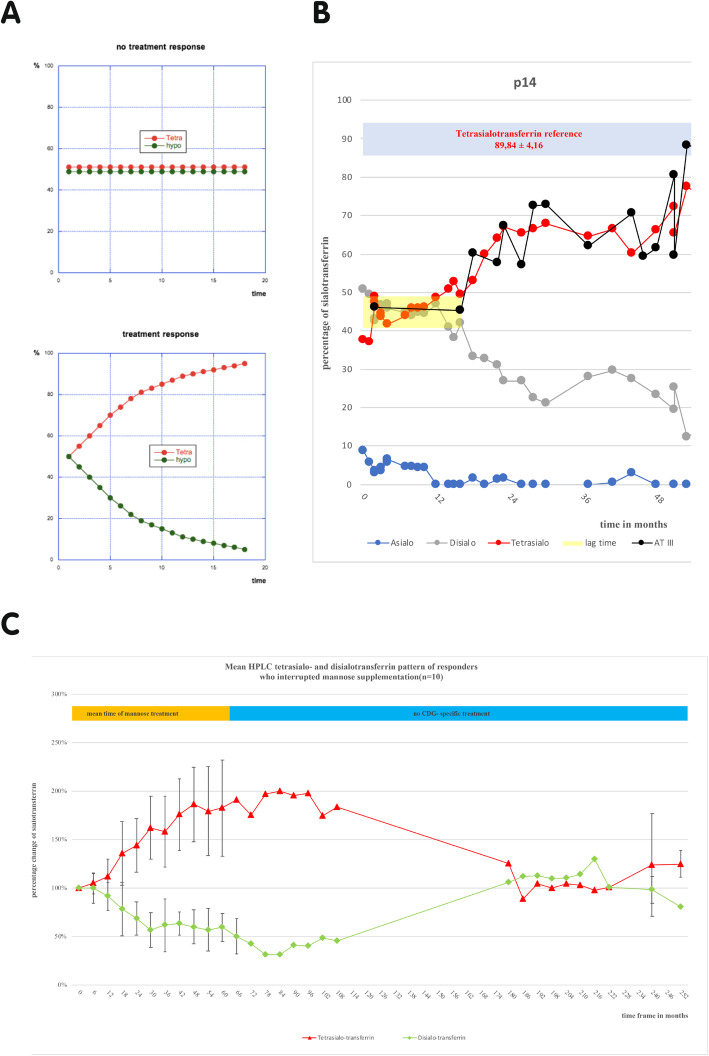
**a** Hypothetical scheme showing expected curves with response or non-response to mannose treatment. **b** Many responders showed a lag time of fluctuating sialo-transferrin values before response (mean 21 ± 8 months). The graph shows the sialo-transferrin-HPLC pattern during mannose supplementation of an index patient. Red line represents tetrasialo-transferrin, green line represents hypoglycosylated disialo-transferrin. This patient showed physiological fluctuations under mannose treatment for approximately 17 months, before hypoglycosylation improved (yellow area). The example of AT III (black graph) shows, that other glycoproteins like coagulation parameters reacted concordantly to the sialo-transferrin pattern. The blue area represents the physiological value of tetrasialo-transferrin. **c** Graph showing the percentage change of tetra- (red graph) and disialo-transferrin (green graph) quantified by HPLC of the responding patients. The initial pretreatment values of tetrasialo- and disialo-transferrin were defined as 100%. The sialo-transferrin values measured during mannose supplementation were set in relation to the initial value to show the follow up. Under mannose therapy tetra- and disialo-transferrin values improved steadily to twice the initial value. After cessation of mannose treatment (orange mark), the sialo-transferrin rates declined to nearly pretreatment values (*n* = 10)

Before mannose therapy, all patients had an abnormal glycosylation pattern in isoelectric focusing (IEF, *data not shown*) and high-performance liquid chromatography (HPLC) of serum transferrin. Sialo-transferrin glycosylation improved considerably in 12 patients under mannose (Fig. 1c, S2). Other glycoproteins such as Antithrombin III, Protein C, Protein S and Factor XI as well as transaminases improved to the same extent as the transferrin- HPLC pattern (Fig. 1b, *not all data shown*).

Two responders were still treated with mannose at the time the data was collected (Fig. S2).

Ten probands with a response to mannose supplementation stopped mannose supplementation after respectively different periods of time (Fig. 1c). Four patients, who stopped mannose supplementation are still undergoing follow up for a mean period of 43,25 ± 3 months without mannose therapy. Six of these ten patients did not receive any CDG-specific treatment for a mean period of 14,2 ± 1 years. Interruption of mannose intake after this time resulted in the reoccurrence of deficient HPLC glycosylation patterns to pretreatment values (Fig. 1c). No patient was able to reach the reference range of transferrin glycosylation. Non-responders had lower pretreatment tetrasialo-transferrin values than responders (Figure S4). There was no significant relationship between a certain genotype and a response to mannose therapy (Table S1).

### Mannose concentrations

Data of blood mannose concentrations of 14 patients with a mannose dosage of 1 g/kg b.w. were analyzed. The mean D-mannose concentration before ingestion of mannose was 30,65 ± 7,45 μmol/l (Figure S3). Blood mannose concentrations did not reach 250 μmol/l, a value necessary for positive in vitro results [10]. After about 5 h the blood mannose values had returned to pre-ingestion levels.

### Clinical presentation and development

Since this was not a formal prospective study, clinical data were not systematically documented to a degree that would allow detailed analysis of all aspects relevant to PMM2-CDG. Avalaible data were analyzed as described by Schiff et al. [23] and are shown in Table S2. Clinical data were collected at first and last consultations. Data suggest that responders to mannose supplementation showed a better progress compared to non-responders in language, reading, writing and counting skills. Higher initial tetrasialo-transferrin values suggested a better response to mannose therapy (Figure S4). No significant correlation between clinical development under mannose therapy and genotype was found.

**Fig. 2:**
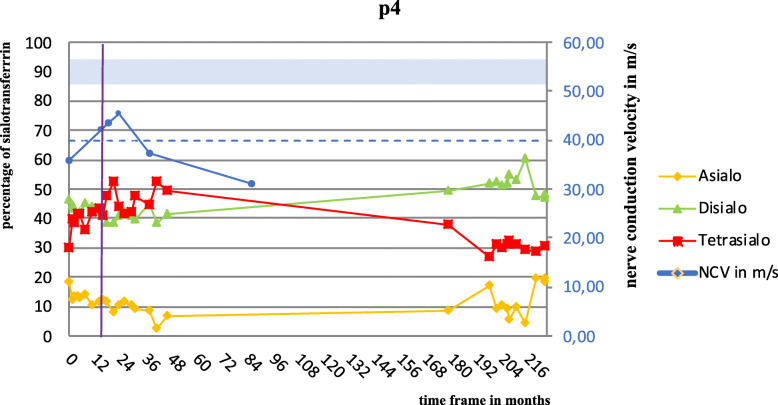
One patient’s pretreatment NCV (nerve conduction velocity) of the posterior tibial nerve improved and stayed stable within 2 years of mannose treatment (NCV reference of posterior tibial nerve > 40 m/s; blue dashed line; find nerve conduction velocity scale on the right in m/s). Improvement of the sialo-transferrin pattern was concordant to NCV development (blue area = tetrasialo-transferrin reference; find sialo-transferrin scale in % on the left). When mannose treatment was discontinued for this patient after 24 months (purple mark), the NCV of the posterior tibial nerve fell and stayed low for four more years without mannose

In PMM2 deficiency, nerve conduction velocity as well as peripheral tendon reflexes gradually deteriorate with time [3, 17, 23–25]. Three responders with follow up data showed a normalization of their nerve conduction velocity (NCV) during mannose therapy (reference values: ulnar nerve: > 49 m/s, Post. tibial nerve: > 40 m/s) (Fig. 2, *not all data shown*). Four responders who already had lost proprioceptive reflexes at first clinical examination had a re-occurrence of proprioceptive reflexes (particularly knee jerk).

## Discussion

Current research in PMM2-CDG tries to increase the flux of Man-6-P (mannose-6-phosphate) into the glycosylation pathway by pharmaceutical means or by mannose derivatives [6]. Mannose was not considered as a potential therapy for PMM2-CDG in recent years. This first approach of a long-term mannose supplementation (≥ 2 years) in PMM2-CDG provides data to show that mannose is not completely inert in PMM2-CDG and suggests that the underrecognized role of mannose in PMM2-CDG should be reconsidered. Long-term oral mannose supplementation in PMM2-CDG was well tolerated, led to biochemical improvements in the majority of patients and suggests possible clinical improvements (Figs. 1, 2, S2).

Whereas mannose supplementation is the standard of care treatment for MPI-CDG resulting in favorable effects on the biochemistry and the clinical outcome [5, 7], short-term dietary supplementations in PMM2-CDG with mannose at 100 mg/kg b.w. every 3 h over 9 days [12] or 0,17 g/kg b.w. every 3,5 h over a period of 6 months [14], as well as a continuous i.v. mannose infusion of 5,7 g/kg b.w. over a period of 3 weeks [13] failed to show improvements in glycosylation patterns or clinical benefits. Mannose therapy in vivo could fail because of Man-6-P being catabolized by the fully operative MPI, transferring the surplus of mannose to glycolysis [6]. In vitro, the glycosylation deficiency in PMM2-deficient fibroblasts can be restored by supplementing more than 250 μmol/l mannose [10, 14]. Assuming, that mutations in PMM2 are hypomorph and reduce enzyme affinity for Man-6-P [9], it is possible, that exogenous mannose supplementation in vitro leads to an increased intracellular Man-6-P concentration, that counters the increased K_m_ requirements of deficient PMM2 fibroblasts. This may lead to higher levels of Man-1-P, increasing the deficient GDP-mannose pools and culminating in normalization of glycosylation [15]. Another possibility may be cytosolic mannose being directly converted into Man-1-P by another enzyme or system (not detected yet) [9].

In vivo, mannose therapy needed a long time to show effects in PMM2-CDG patients. Even in MPI-CDG, the first partial corrections in IEF- and SDS- patterns of serum transferrin needed several to occur after initiation of a mannose therapy with a dose of 100 mg/kg three times a day [5]. This cannot be explained by the biological half-life of transferrin (CDT = ~14d; Non-CDT = ~8d) and other glycoproteins (AT III: 3d; Protein C: 6-8 h) [16]. Effects on the IEF pattern would be expected not later than 4 weeks. A biochemical correction in PMM2-CDG may take a longer time and higher mannose dosage to show positive effects, in the light of countering the K_m_- requirement of the attenuated PMM2. Further investigations of mannose pools used for N-glycosylation might explain this time-lag in the future. Ichikawa et al. found that the contribution of exogenous mannose to N-glycosylation is higher than previously thought and that other potential sources of mannose such as mannose salvaged from degraded glycoproteins, glycogen and gluconeogenesis do not make significant contributions [8]. In fibroblasts, increased exogenous mannose (1 mM) can completely replace glucose-derived mannose and become the sole source of mannose in N-glycans and also contribute to galactose and N-acetylglucosamine in N-glycans [8, 26]. Explanations for the higher contribution of mannose to N-glycans may be specific mannose transporters (GLUT-like mannose transporter, SGLT-5 mannose specific transporter) [27, 28]. About one third of mannose found in N-glycans takes detours as it is first converted to Frc-6-P and reconverted to Man-6-P again. Since the transient Frc-6-P derived from Man-6-P does not equilibrate with the total cellular pool of Frc-6-P, another suggestion may be the presence of separate Frc-6-P-pools (Frc-6-P^GP^/Frc-6-P^G^) like check-points for glycosylation, glycolysis and gluconeogenesis, generated by the anomeric selectivity of Glc-6-P and Man-6-P metabolizing enzymes (Fig. S1) [8, 26]. A preferable ratio of α- and ß- Man-6-P as well as of MPI (ß-Man-6-P anomer specific) and PMM2 (α-Man-6-P anomer specific) might result in a higher efficiency of exogenous mannose use in glycosylation [8]. Substances enhancing the impact of check points of mannose flux to the glycosylation pathway may improve the effect of exogenous mannose supplementation. Which other undefined factors and check points of mannose metabolism [8] have an influence on the effect of mannose supplementation needs to be further investigated.

It has to be considered, that the glycosylation of transferrin and other glycoproteins may improve in time, with age and the degree of liver involvement [23, 24, 29]. There are 2 major arguments against spontaneous improvement in this study. First, the patients in this study started mannose therapy at very different ages from 1 year to 27 years of age (Table S1). Nevertheless, they showed a similar improvement with similar kinetics (Fig. 1, S2), suggesting that mannose supplementation, not age, was responsible for glycosylation improvement. Secondly, the significant correction of the hypoglycosylated serum transferrin returned to approximately pretreatment patterns after long-term interruption of mannose supplementation (Fig. 1c). This clearly indicates the biochemical effect of mannose on glycosylation and not an improvement with age.

Some patients stopped mannose therapy. Giving mannose with meals 5 times a day is time consuming for parents and guardians. Patients might experience episodes of flatulence or diarrhea, which can make an already seriously ill child even more uncomfortable. Mannose dosages have to be slowly increased over weeks and mannose has to be given with food in order to decrease these problems.

Repetitive doses of orally ingested mannose can maintain elevated blood mannose levels in PMM2-CDG patients [30]. Healthy probands reached blood mannose levels of more than 200 μmol/l after 1 h by supplementing 0.2 g mannose/kg b.w [26, 27]. The patients in this study showed fluctuating blood mannose with an average blood mannose concentration not reaching the level of 250 μmol/l. With 1 g/kg b.w. mannose per day, blood mannose levels could not be maintained properly over the daily period and night time and were lower on average than the concentration shown to correct abnormal glycosylation in fibroblasts (250 μmol/l) [10, 14]. If one assumes that an increased mannose concentration counters the K_m_- requirements of deficient PMM2, a constantly increased blood mannose concentration might be more essential, than peak values in order to constantly provide sufficient mannose donors for N- and C- glycosylation. Despite the positive responses in the majority of patients, these circumstances have certainly limited the effectiveness of mannose. Parenteral application, for instance by subcutaneous infusion, might be an approach to reach steady blood mannose levels during day and night time.

Since the collection of the clinical data in this study was done in everyday clinical practice without a specific protocol and without matching a control group, retrospective analysis necessarily introduces some bias, which affect the interpretation regarding outcome and coherence negatively. The findings prove a biochemical effect and suggest a clinical effect of mannose therapy. Current literature gives no indication for a normalization of nerve conduction velocity in patients with PMM2-CDG. This study observed PMM2-CDG patients with mannose therapy developing a normalization of their motor nerve conduction velocity and regaining a redeemable knee jerk. Parents and caretakers uniformly reported improved reactivity, attention and better general state while supplementing mannose. The clinical data in this study give the impression that responders with higher initial tetrasialo-transferrin values (Figure S4) and milder phenotype tended to show an improved clinical development. We could not find a significant relation between a certain genotype and a response to mannose therapy.

No therapy will reverse all symptoms of the disease. There are crucial developmental steps during pregnancy and early childhood being disrupted and leading to evident, irreversible malformations and abnormalities (similar to MPI-patients with ductal plate malformations in the liver [31]). Different organ manifestations of PMM2-CDG may have differently effective responses to a mannose treatment, due to crucial developmental steps during embryogenesis and infancy being negatively affected by hypoglycosylation [11]. Therefore, very early or even prenatal therapy may be an issue to make a significant difference in improving these children’s health condition [32]. Detailed clinical efficacy of dietary D-mannose in PMM2-CDG should be tested in a controlled, double blinded, randomized study.

## Conclusion

This study demonstrates mannose as one effective cornerstone in a therapeutic approach for PMM2-CDG. It was demonstrated that mannose has a biochemical impact on a majority of PMM2-CDG patients. The effect of mannose is depending on the PMM2:MPI ratio and the anomeric preference of PMM2 and MPI [8, 15, 26] and may be improved by co-administration of pharmaceuticals such as PMM2-activators, MPI inhibitors, PMM2-replacement [15, 33], pharmacological chaperoning [34], proteostasis regulators [35] and others. This may reduce mannose side effects and improve the metabolic flux of mannose into glycosylation. Mannose had no severe side effects even in long term supplementation. Possible confounding factors include impaired intestinal mannose absorption as well as a fluctuating and insufficient serum mannose concentration. Thus, this study should promote viable animal models together with controlled clinical approaches (especially double-blinded randomized trials) to further evaluate the therapeutic potential of mannose in PMM2-CDG and to further test simultaneous approaches with the aforementioned perspectives.

## Supplementary information

**Additional file 1: Figure S1.** Layout showing the metabolic fate of mannose in mammalian cells. **PMM2** is an essential enzyme catalyzing the conversion of mannose-6-phosphate to mannose-1- phosphate, which is the first step in the synthesis. **Figure S2.** Example of two patients, who did not stop the mannose supplementation and were still treated with mannose at the time the data was collected. Sialo-transferrin values quantified by HPLC during mannose supplementation. **Figure S3.** Mean blood mannose concentration obtained after every half hour from 30-300min after mannose ingestion of 1g/kg BW on clinical consultations. **Figure S4.** Boxplots comparing the initial tetrasialo-transferrin values before mannose therapy in responders and in non-responders. **Table S1.** Patients with mannose therapy reported in this study. **Table S2.** Clinical findings in all patients and by group (responders vs. non-responders).

## Data Availability

Not applicable.
