# Supplementary material for: Dietary mannose supplementation in phosphomannomutase 2 deficiency (PMM2-CDG)
Source: Orphanet J Rare Dis. 2020 Sep 22;15:258. doi: 10.1186/s13023-020-01528-z (PMC7510076; doi:10.1186/s13023-020-01528-z)
Supplement: Supplementary file 1 — Additional file 1: Figure S1. Layout showing the metabolic fate of mannose in mammalian cells. PMM2 is an essential enzyme catalyzing the conversion of mannose-6-phosphate to mannose-1- phosphate, which is the first step in the synthesis. Figure S2. Example of two patients, who did not stop the mannose supplementation and were still treated with mannose at the time the data was collected. Sialo-transferrin values quantified by HPLC during mannose supplementation. Figure S3. Mean blood mannose concentration obtained after every half hour from 30-300min after mannose ingestion of 1g/kg BW on clinical consultations. Figure S4. Boxplots comparing the initial tetrasialo-transferrin values before mannose therapy in responders and in non-responders. Table S1. Patients with mannose therapy reported in this study. Table S2. Clinical findings in all patients and by group (responders vs. non-responders). [file 13023_2020_1528_MOESM1_ESM.docx]

**Dietary mannose supplementation in phosphomannomutase 2 deficiency (PMM2-CDG)**

Roman Taday^1^, Marianne Grüneberg^1^, Ingrid DuChesne^1^, Janine Reunert^1^,

Thorsten Marquardt^1^

^1^University Children’s Hospital Münster, Department of General Pediatrics, Münster, Germany

**Supplements**

**
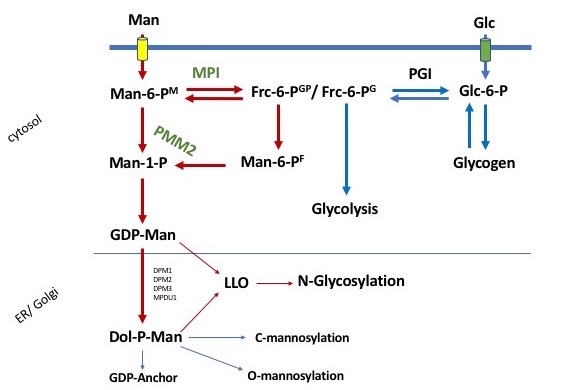
**

**Figure S1:** Layout showing the metabolic fate of mannose in mammalian cells. PMM2 is an essential enzyme catalyzing the conversion of mannose-6-phosphate to mannose-1- phosphate, which is the first step in the synthesis

of glycosylation intermediates including GDG mannose, GDP-fucose and dolichol-P-mannose. These activated

mannose donors are required for N- and O- glycosylation, the biosynthesis of glycophosphotidylinositol anchors

and C- mannosylation. PMM2 deficiency results in reduced amounts of Man-1-P and Man-6-P and unoccupied N

linked glycosylation sites on a number of proteins. [1] Substances enhancing the impact of check points (green

box) of glycosylation flux may improve the effect of exogenous mannose supplementation.

**A)**

**B)**

**
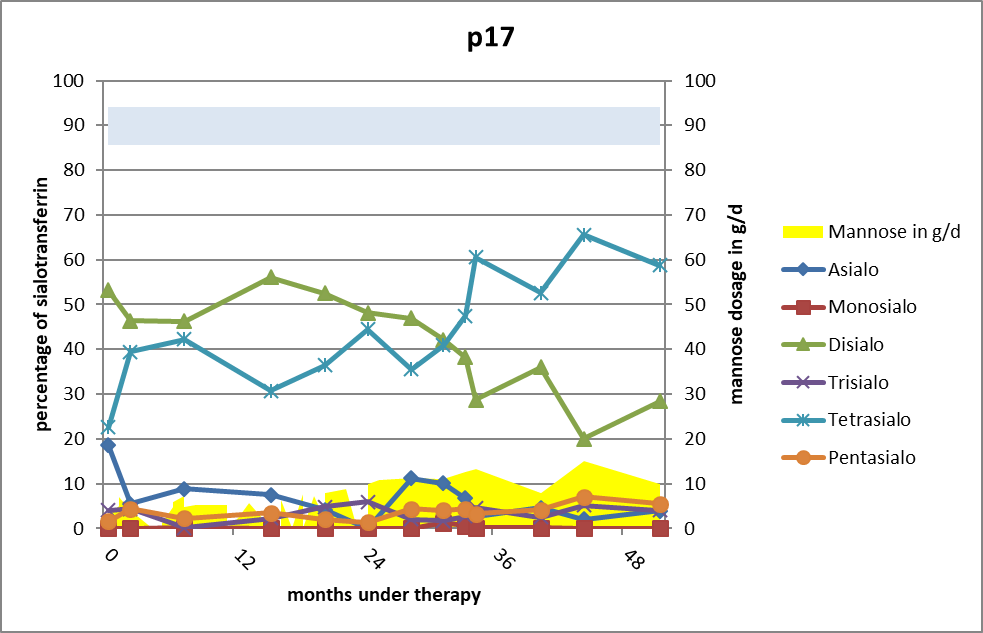
**

**Figure S2:** Example of two patients, who did not stop the mannose supplementation and were still treated with mannose at the time the data was collected. Sialo-transferrin values quantified by HPLC during mannose supplementation. Tetrasialo-Tf reference is labelled in blue area. Time under mannose supplementation shows a rather rapid increase in tetrasialo-transferrin with a simultaneous decrease in evenly hypoglycosylated sialo-transferrin proteins. We found a lag- time of stagnating values, before we have a rather rapid increase of the sialo-transferrin pattern.

**Figure S3:** Blood samples were obtained at 30, 60, 90, 120 150, 180, 210, 240 and 300 min after mannose ingestion on clinical consultations. 14 patients provided utilizable data with a mannose dosage of 1g/kg BW. The blue graph shows the mean blood mannose concentration at the respective points in time ±SD. The red line represents the value of 250 µmol/l of mannose, which showed positive results in vitro [2].


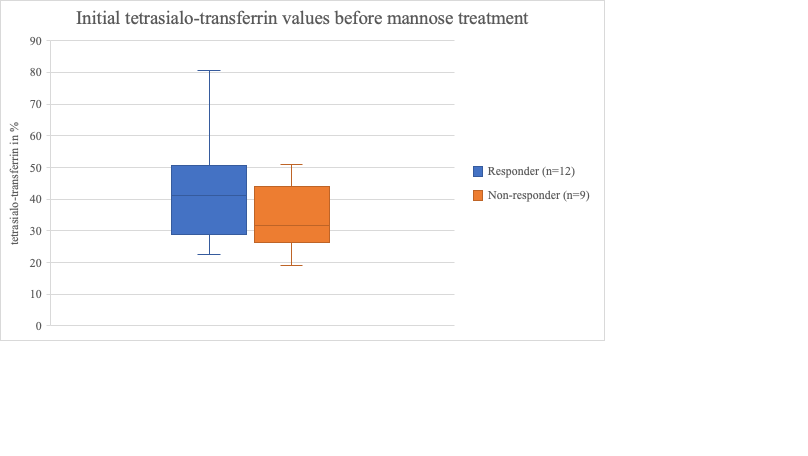


**Figure S4:** These boxplots compare the initial tetrasialo-transferrin values before mannose therapy in responders and in non-responders. Half of the responders (median responders: 41,01%) had a higher pretreatment tetrasialo-transferrin than half of the non-responders (median non-responders: 31,82%). Higher initial tetrasialo-transferrin values before mannose therapy seem to be associated with a better response to mannose therapy.

**Table S1: Patients with mannose therapy reported in this study**

| **Patient** | **Age at start of mannose therapy** | **Mutation**  RefSeq NM_000303.3  (PMM2)  NP_000294.1 | **duration of therapy** | **max. mannose dose in g/d** | **pretreatment tetrasialo-transferrin value**  **in %** | **max. tetrasialo-transferrin value under therapy**  **in %** | **reason for supplementation stop** |
| --- | --- | --- | --- | --- | --- | --- | --- |
| **Responder** |  |  |  |  |  |  |  |
| **p1** | 4 years 1 month | c.{422G>A} (p.Arg141His) [R141H]+  c.{686A>C} (p.Tyr229Ser) [Y229S] | 145 months | 66,5 | 44,77 | 83,7 | Still under mannose supplementation. |
| **p4** | 6 years 11 months | c.{357C>A} (p.Phe119Leu) [F119L]+  c.{422G>A} (p.Arg141His) [R141H] | 36 months | 28 | 30,04 | 55,24 | No further mannose supply after initial study. Restarted at the age of 23 years. |
| **p7** | 1 year | c.{377T>C} (p.Met126Thr) [M126T]+  c.{422G>A} (p.Arg141His) [R141H | 38 months | 43 | 69,1 | 87,7 | Patient moved to other country and did not continue supplementation. |
| **p9** | 3 years 8 months | c.{357C>A} (p.Phe119Leu) [F119L]+  c.{422G>A} (p.Arg141His) [R141H] | 53 months | 15 | 25,54 | 65,62 | Stopped due to unfavourable opinions about mannose in PMM2-CDG at that time. |
| **p10** | 8 years | c.{377T>C} (p.Met126Thr) [M126T]+  c.{422G>A} (p.Arg141His) [R141H | 66 months | 38 | 51,42 | 75,75 | Stopped due to unfavourable opinions about mannose in PMM2-CDG at that time. |
| **p12** | 2 years | c.{338C>T} (p.Pro113Leu) [P113L]+  c.{422G>A} (p.Arg141His) [R141H | 73 months | 32 | 27,12 | 66,01 | The care of the patient proved to be too burdensome for the parents. |
| **p13** | 1 year 8 months | c.{470T>C} (p.Phe157Ser) [F157S] +  c.{710C>T} (p.Thr237Met) [T237M] | 73 months | 54 | 29,49 | 56,4 | Patient moved to other country and did not continue supplementation |
| **p14** | 8 months | c.{640-15479C>T} [IVS7+19138]  c.{667G>A} (p.Asp223Asn) [D223H] | 48 months | 37 | 37,62 | 77,51 | unknown |
| **p16** | 1 year | c.{422G>A} (p.Arg141His) [R141H]+  c.{338C>T} (p.Pro113Leu) [P113L] | 65 months | 23 | 50,56 | 75,41 | Stopped due to unfavourable opinions about mannose in PMM2-CDG at that time |
| **p17** | 1year | c.{357C>A} (p.Phe119Leu) [F119L]+  c.{422G>A} (p.Arg141His) [R141H] | 44 months | 15 | 22,57 | 65,6 | Still under mannose supplementation. |
| **p19** | 1 year | c.{422G>A} (p.Arg141His) [R141H]+  c.{640-9T>A} [IVS7-9T>A] | 24 months | 9 | 44,41 | 70,04 | Gastrointestinal side effects. |
| **p20** | 5 years 2 months | c.{199G>A}(p.Val67Met) [V48M]* +  c.{422G>A} (p.Arg141His) [R141H] | 45 months | 22 | 40,19 | 72,86 | Stopped due to unfavourable opinions about mannose in PMM2-CDG at that time |
| **Non-Responder** |  |  |  |  |  |  |  |
| **p2** | 27 years | c.{357C>A} (p.Phe119Leu) [F119L]+  c.{669C>G} (p.Asp223Glu) [D223E] | 42 months | 65 | 51,08 | 53,12 | Still under mannose supplementation |
| **p3** | 11 years 11 months | c.{357C>A} (p.Phe119Leu) [F119L]+  c.{422G>A} (p.Arg141His) [R141H] | 44 months | 50 | 23,86 | 33,34 | Still under mannos supplementation |
| **p5** | 3 years 7 months | c.{422G>A} (p.Arg141His) [R141H]+  c.{663C>G}(p.Phe221Leu) [F221L] | 45 months | 26 | 44,62 | 53,39 | Still under mannose supplementation |
| **p6** | 2 years | c.{338C>T} (p.Pro113Leu) [P113L]+  c.{422G>A} (p.Arg141His) [R141H] | 47 months | 22,5 | 27,02 | 35,36 | Still under mannose supplementation |
| **p8** | 1 year | c.{357C>A} (p.Phe119Leu) [F119L]+  c.{357C>A} (p.Phe119Leu) [F119L] | 79 months | 24 | 27,01 | 32,52 | Still under mannose supplementation |
| **p11** | 17 years | c.{422G>A} (p.Arg141His) [R141H]+  c.{677C>G} (p.Thr226Ser) [T226S] | 45 months | 60 | 35,94 | 46,01 | Still under mannose supplementation |
| **p15** | 2 years | c.{422G>A} (p.Arg141His) [R141H]+  c. {531 G>C}(p.Gln177His) [Q177H] | 84 months | 26 | 43,68 | 44,44 | Still under mannose supplementation |
| **p18** | 9 months | c.{422G>A} (p.Arg141His) [R141H]+  c.{710C>T} (p.Thr237Met) [T237M] | 59 months | 18,5 | 27,95 | 38,45 | not known |

**Table S1:** This table provides background information on the patients included in this study divided into responders and non-responders. The mean PMM2-activity = 3,625% ± 4,6% (n=8). Median age of mannose therapy start of responders: 1,85 years. Median age of non-responders: 2,8 years.

* RefSeq: NG_009209.1:g.11975G>A

**Table S2: Clinical findings in all patients and by group (responders vs. non-responders)**

| **Characteristics**  **(n=number of patients with available data)** | **All patients** | **Responder** | **Non-**  **responder** |
| --- | --- | --- | --- |
| **Failure to thrive (n=20)** | 20 (100%) | 12 (60%) | 8 (40%) |
| **Dysmorphic symptoms (n=13)** | 12 (92,3%) | 9 (75%) | 3 (25%) |
| **Motor involvement (n=20)** | 20 (100%) | 12 (60%) | 8 (40%) |
| **Axial hypotonia (n=13)** | 13 (100%) | 10 (76,9%) | 3 (23,1%) |
| **Cerebellar hypoplasia (n=10)** | 9 (90%) | 4 (44,4%) | 5 (55,6%) |
| **Ataxia (n=12)** | 12 (100%) | 8 (66,7%) | 4 (33,3%) |
| **Stroke-like episodes (n=5)** | 4 (80%) | 2 (50%) | 2 (50%) |
| **Epilepsia (n=6)** | 5 (83,3%) | 4 (80%) | 1 (20%) |
| **Hydrocele (n=1)** | 1 (100%) | 1 (100%) | 0 (0%) |
| **Upper limb nerve conduction velocitiy reduced (n=1)** | 1 (100%) | 1 (100%) | 0 (0%) |
| **Lower limb nerve conduction velocity reduced (n=10)** | 10 (100%) | 8 (80%) | 2 (20%) |
| **No proprioceptive reflex (n=17)** | 14 (82,35%) | 9 (64,3%) | 5 (35,7%) |
| **Positive Babinski sign (n=12)** | 3 (25%) | 2 (66,7%) | 1 (33,3%) |
| **Cognitive issues (n=5)** | 5 (100%) | 4 (80%) | 1 (20%) |
| **Reading before 10 years (n=2)** | 2 (100%) | **2 (100%)** | 0 (0%) |
| **Aquired (n=1)** | 1 (100%) | **1 (100%)** | 0 (0%) |
| **Able to decipher (n=2)** | 2 (100%) | **2 (100%)** | 0 (0%) |
| **Counting (n=5)** | 5 (100%) | **3 (60%)** | 2 (40% |
| **Writing (n=5)** | 3 (60%) | **2 (66,7%)** | 1 (33,3%) |
| **Aquired (n=2)** | 1 (50%) | 0 (0%) | 1 (100%) |
| **Single words (n=3)** | 3 (100%) | **2 (66,7%)** | 1 (33,3%) |
| **Speaks words before 2 years (n=10)** | 6 (60%) | **4 (66,7%)** | 2 (33,3%) |
| **Speaks 3 word sentences**  **before 5 years (n=10)** | 9 (90%) | **7 (77,8%)** | 2 (22,2%) |
| **Speaks sentences**  **before 10 years ≥ 2 years (n=4)** | 2 (50%) | 2 (100%) | 0 (0%) |
| **Adapted education in normal school or kindergarden (n=7)** | 7 (100%) | 4 (57,1%) | 3 (42,9%) |
| **Special institution for disabled individuals (n=7)** | 7 (100%) | 3 (42,9%) | 4 (57,1%) |
| **Free sitting before 2 years (n=15)** | 7 (46,7%) | **6 (85,7%)** | 1 (14,3%) |
| **Standing with help device /AID**  **before 2 years (n=13)** | 4 (30,8%) | **4 (100%)** | 0 (0%) |
| **Standing without help**  **in ≥2-10 years (n=10)** | 8 (80%) | **5 (62,5%)** | 3 (37,5%) |
| **Walking autonomously**  **before 2 years (n=16)** | 0 (0%) | 0 (0%) | 0 (0%) |
| **Walking with help device/AID**  **before 2 years (n=12)** | 1 (8,3%) | **1 (100%)** | 0 (0%) |
| **Walking autonomously**  **in ≥2- 10 years (n=7)** | 1 (14,3%) | **1 (100%)** | 0 (0%) |
| **Walking with help device/aid before ≥2- 5 years (n=15)** | 12 (80%) | **8 (66,7%)** | 4 (33,3%) |
| **Sensorineural deafness (n=9)** | 0 (0%) | 0 (0%) | 0 (0%) |
| **Strabismus (n=15)** | 14 (93,3%) | 9 (64,3%) | 5 (35,7%) |
| **Retinitis pigmentosa (n=5)** | 1 (20%) | 0 (0%) | 1 (100%) |
| **Nystagmus (n=6)** | 3 (50%) | 1 (33,3%) | 2 (66,7%) |
| **Astigmatism (n=4)** | 1 (25%) | 1 (100%) | 0 (0%) |
| **Myopia (n=2)** | 2 (100%) | 1 (50%) | 1 (50%) |
| **Hyperopia (n=4)** | 4 (100%) | 2 (50%) | 2 (50%) |
| **Kyphosis and scoliosis (n=8)** | 4 (50%) | 3 (75%) | 1 (25%) |
| **Osteopenia (n=3)** | 0 (0%) | 0 (0%) | 0 (0%) |
| **Thorax deformation (n=5)** | 2 (40%) | 2 (100%) | 0 (0%) |

**Table S2:** Categories were used as in Schiff et al. 2018 [3]. The number of patients with available data is reported between brackets next to the item. First column presents the absolute number of patients with the percentages of patients out of the number of available data. Second and third column present the number of patients divided as per response to mannose. Between brackets are the percentages of patients out of the number of all patients who presented the considered item (first column).

**References**

1. Ichikawa, M.; Scott, D.A.; Losfeld, M.-E.; Freeze, H.H. The metabolic origins of mannose in glycoproteins. *J. Biol. Chem.* **2014**, *289*, 6751–6761, doi:10.1074/jbc.M113.544064.

2. Panneerselvam, K.; Freeze, H.H. Mannose corrects altered N-glycosylation in carbohydrate-deficient glycoprotein syndrome fibroblasts. *J. Clin. Invest.* **1996**, *97*, 1478–1487, doi:10.1172/JCI118570.

3. Schiff, M.; Roda, C.; Monin, M.-L.; Arion, A.; Barth, M.; Bednarek, N.; Bidet, M.; Bloch, C.; Boddaert, N.; Borgel, D.; et al. Clinical, laboratory and molecular findings and long-term follow-up data in 96 French patients with PMM2-CDG (phosphomannomutase 2-congenital disorder of glycosylation) and review of the literature. *J. Med. Genet.* **2017**, doi:10.1136/jmedgenet-2017-104903.
